# Safety and efficacy of endoscopic sleeve gastroplasty after liver transplantation

**DOI:** 10.1016/j.igie.2025.03.013

**Published:** 2025-04-04

**Authors:** Benjamin I. Richter, Yazan Abboud, Jay Phansalkar, Keri E. Lunsford, Zaid Tafesh, Arpit Amin, Dhvani Dosh, Ahmed Al-Khazraji, Suut Gokturk, Paul Gaglio, Kaveh Hajifathalian

**Affiliations:** 1 Department of Gastroenterology, Newark, New Jersey; 2 Department of Medicine, Newark, New Jersey; 3 Division of Transplant and HPB Surgery, Department of Surgery, Newark, New Jersey; 4 Department of Transplant Hepatology, Newark, New Jersey; 5 Rutgers New Jersey Medical School, Newark, New Jersey

## Abstract

**Background and Aims:**

Obesity after liver transplantation (LT) can compromise patient outcomes. We report safety and efficacy data on endoscopic sleeve gastroplasty (ESG) after LT.

**Methods:**

This is a retrospective case series of patients who underwent ESG after LT.

**Results:**

All 5 patients underwent ESG at a median 47.3 months (interquartile range [IQR], 34.4-67.4 months) after LT. Patients were predominantly male (4/5, 80%) with median age 61 years (IQR, 51-61 years) and preoperative body mass index 38.8 kg/m^2^ (IQR, 37.1-42.6 kg/m^2^). Median follow-up after ESG was 18.8 months (IQR, 8.2-19.8 months). Median total body weight loss was 27.2% (IQR, 8.0%-30.0%) at the end of follow-up. Weight loss plateau occurred in 2 patients, with 5 postoperative adverse events in 4 patients (80%) that were mild, and none exceeded grade I on the Clavien-Dindo scale. No patient missed doses of immunosuppressive medications. Liver graft function remained stable.

**Conclusions:**

Our data support the use of ESG after LT, although more data are needed to validate our findings.

Obesity is common after liver transplantation (LT); contributors include dietary changes, underlying metabolic syndrome, and post-LT medications.^1^ Obesity after LT is associated with a 2-fold increased mortality risk, and body mass index (BMI) is inversely associated with long-term survival.^2^ Metabolic dysfunction–associated steatotic liver disease (MASLD) recurrence is common after LT, primarily driven by obesity, and post-LT steatosis occurs with higher frequency and severity in patients with pre-existing MASLD.^3^ Weight control after LT is therefore essential for favorable outcomes.

Metabolic surgery remains the most effective modality for treating obesity, but its use is limited in post-LT patients by increased overall mortality, postoperative adverse events, and hospitalization costs.^4^ Endoscopic sleeve gastroplasty (ESG) is a minimally invasive intervention in which full-thickness sutures are placed endoscopically across the stomach to reduce gastric volume. It is associated with a serious adverse event rate of 1.25%, a mean total body weight loss (TBWL) of 15.66% at 12 months with sustained long-term weight loss reported, and improvements in multiple metabolic parameters and obesity-related comorbidities.^5^^,^^6^ Emerging data have also highlighted the potential impact of ESG on MASLD. A recent meta-analysis found that ESG is associated with improvements in multiple liver parameters,^7^ and a trial comparing ESG plus lifestyle modification with sham endoscopy corroborated these hepatic improvements, reinforcing the role for ESG in MASLD management.^8^ Here, we report our data on the safety and efficacy of ESG after LT.

## Methods

### Patient population and definitions

This retrospective case series was approved by our Institutional Review Board with waiver of informed consent (IRB protocol Pro2023002422). Among 79 patients who underwent endobariatric procedures from December 2021 to May 2024, 5 patients underwent ESG after LT, all of whom were included in our study.

Weight regain was defined as ≥50% weight gain compared with the patient’s nadir weight loss after ESG. Weight loss plateau was defined as weight loss of ≥10% TBWL without significant additional weight loss within the first year post-ESG or inadequate weight loss (failure to achieve ≥10% TBWL after ESG).

Patients were referred for endobariatrics by Transplant Hepatology. Indications for ESG included at least class I obesity (BMI ≥30 kg/m^2^) or overweight (BMI ≥27 kg/m^2^) with obesity-associated comorbidities and unsuccessful weight loss with noninvasive measures such as exercise, dietary modifications with nutritionist support, and, in most cases, anti-obesity medication (AOM) use. Patients were evaluated in a multidisciplinary obesity clinic, where treatment options—including noninvasive measures, metabolic surgery, and ESG—were discussed. Contraindications for ESG included cancer at the time of procedure, history of gastric cancer, end-stage organ disease, pregnancy, and severe or untreated mental illness. Liver biopsies were performed on all patients at some point before ESG based on our institution’s protocol. Post-transplantation liver biopsies were also performed per protocol to assess for fibrosis in patients at risk for MASLD-induced fibrosis.

### Study treatments

**Figure 1:**
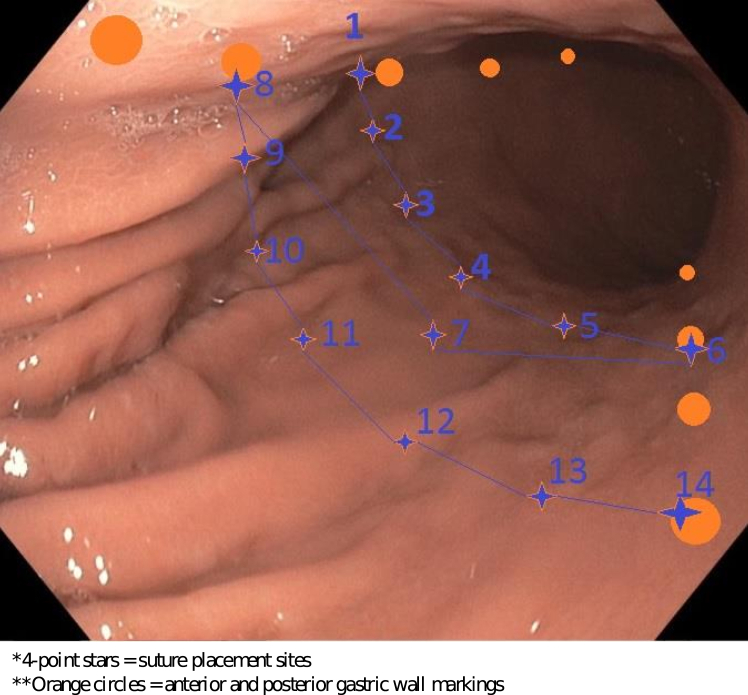
Endoscopic view of stomach body with *orange circles* showing spots for argon plasma coagulation marking and *blue stars* showing placement of sutures in Z-pattern.

The ESG techniques we used were previously described,^9^ with variations detailed below. A single endoscopist (K.H.) performed all procedures according to a standardized protocol in an outpatient setting with the patients under general anesthesia in supine position. Pre-procedural anti-emetics and antibiotics were administered, and baseline immunosuppression was maintained. Essophagogastroduodenoscopy (GIF-H190; Olympus Corp, Tokyo, Japan) was performed before ESG to clear the stomach of debris, measure the distance between the gastroesophageal junction and pyloric sphincter, and mark parallel anterior and posterior suture placement sites by means of argon plasma coagulation from the incisura to cardia. A double-channel endoscope (GIF-2TH180; Olympus Corp) with an affixed OverStitch suturing device (Boston Scientific, Marlborough, Mass, USA) was then used to place full-thickness sutures (Fig. 1). The suturing system includes an actuating handle, needle driver, and anchor-exchange catheter. ESG was created by placing 6 to 8 running full-thickness sutures in a Z-pattern along the anterior wall, greater curvature, and posterior wall, with each suture incorporating 12 to 14 bites, and a second suture layer added to further reduce the reconfigured lumen.

After a standard period of post-anesthesia monitoring, patients were discharged with a 5-day course of prophylactic antibiotics, anti-emetics, proton pump inhibitors, pain medication, and liquid protein shake–based diet with progressive change to solid foods. Follow-up included hepatology and gastroenterology appointments within the first week, with ongoing gastroenterology and nutrition visits every 2 weeks for 3 months and monthly thereafter.

### Outcomes

Data were obtained from electronic health records. The primary clinical outcome was safety, which was measured by the presence and severity of adverse events according to the Clavien-Dindo scale, a validated tool used in multiple ESG studies to characterize adverse events based on the interventions required to manage them.^5^ Tacrolimus trough levels and hepatic function tests were assessed within 1 month after the procedure. Secondary outcomes were TBWL% at end of follow-up and change in AOM use. Data are reported as median (interquartile range [IQR]).

## Results

### Baseline clinical variables

All 5 patients underwent ESG a median of 47.3 months (IQR, 34.4-67.4 months) after LT. Patients were predominantly male (4/5, 80%), with median age 61 years (IQR, 51-61 years) and BMI 38.8 kg/m^2^ (IQR, 37.1-42.6 kg/m^2^) at the time of procedure (Table 1).

**Table 1:** Patient characteristics

Age, y	61 (51-61)
Male sex	4 (80)
Type 2 diabetes mellitus	4 (80)
Hypertension	3 (60)
Etiology of underlying liver disease	
MASLD	1 (20)
ETOH	2 (40)
HCV	1 (20)
Cryptogenic	1 (20)
Pre-LT weight, lb	222 (211-237)
Pre-ESG weight, lb	240 (224-261)
Pre-ESG BMI, kg/m^2^	38.8 (37.1-42.6)
Pre-ESG liver biopsy macrosteatosis, %	5 (0-15)
Pre-ESG Fib-4 score	1.4 (1.2-1.6)
Pre-ESG elastography, kPa	3.6 (3.2-3.7)

Values are reported as n (%) or median (interquartile range).

*BMI*, Body mass index; *ESG*, endoscopic sleeve gastroplasty; *ETOH*, alcohol use; *Fib-4*, Fibrosis-4; *HCV*, hepatitis C virus; *LT*, liver transplantation; *MASLD*, metabolic dysfunction–associated steatotic liver disease.

### Weight loss outcomes

**Figure 2:**
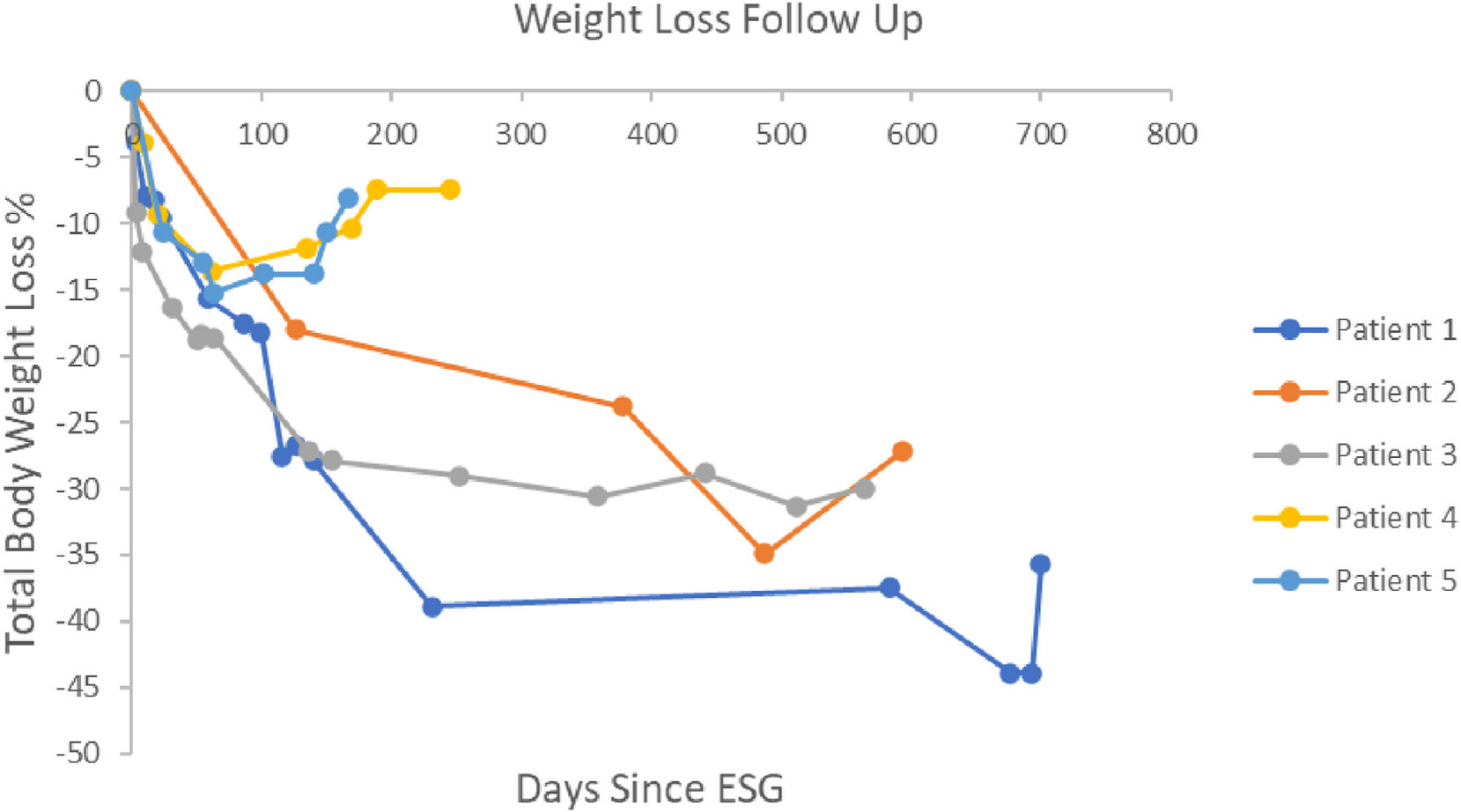
Total body weight loss % over time in days after endoscopic sleeve gastroplasty (ESG) for each patient.

Median follow-up after ESG was 18.8 months (IQR, 8.2-19.8 months). Median TBWL% was 27.2% (IQR, 8.0%-30.0%) at the end of follow-up (Table 2, Fig. 2). Weight loss plateau occurred in 2 patients (cases 4 and 5) (Table 2).

**Table 2:** Outcomes

	Overall	Case 1	Case 2	Case 3	Case 4	Case 5
Weight loss∗						
Follow up, mo	18.8	23.3	19.8	18.8	8.2	5.6
Pre-ESG BMI, kg/m^2^	38.8	42.6	44.8	38.8	31.9	37.1
Pre-ESG weight, lb	240	280	261	240	204	224
Post-ESG nadir weight, lb	170	157	170	165	176	190
End of follow-up weight, lb	189	180	190	168	189	206
Total body weight loss, lb	71	100	71	72	15	18
Total body weight loss, %	27.2	35.7	27.2	30.0	7.4	8.0
Adverse events†						
Mild	5	0	1	2	1	1
Moderate	0	0	0	0	0	0
Severe	0	0	0	0	0	0
Death	0	0	0	0	0	0
Vomiting, d	1	0	3	0	0	0
Missed ISPs, d	0	0	0	0	0	0
Post-ESG admission, d	0	0	1	0	0	0

*BMI*, Body mass index; *ESG*, endoscopic sleeve gastroplasty; *ISPs*, immunosuppressant medications.

∗ Median was reported for weight loss variables in the “Overall” column and as individual case data in the cases 1-5 columns.

† Total adverse events per severity category were reported for all adverse events columns.

### AOM use

Three patients were on glucagon-like peptide-1 receptor agonists (GLP-1RAs) before ESG; 2 discontinued the GLP-1RA (semaglutide and liraglutide) after ESG, and 1 continued (tirzepatide). Two patients used topiramate before ESG, of which 1 discontinued it after ESG owing to sufficient weight loss. Another patient used phentermine-topiramate before ESG and continued it. One patient started using topiramate after ESG (Table 3).

**Table 3:** AOM Use

	Case 1	Case 2	Case 3	Case 4	Case 5
GLP-1 receptor agonists					
Used pre-ESG	Yes∗	Yes†	Yes‡	No	No
Stopped for ESG	Yes	Yes	No	NA	NA
Started post-ESG	No	No	No	No	No
Other AOM use					
Used pre-ESG	No	Yes§	Yes‖	No	Yes¶
Stopped for ESG	N/A	Yes	No	NA	No
Started post-ESG	No	No	No	Yes•	No

*AOM*, Anti-obesity medication; *ESG*, endoscopic sleeve gastroplasty; *GLP-1*, glucagon-like peptide-1; *NA*, not applicable.

∗ Semaglutide, 1 mg subcutaneously once weekly.

† Liraglutide, 3 mg subcutaneously daily.

‡ Tirzepatide, 5 mg subcutaneously once weekly.

§ Topiramate, 25 mg orally daily.

‖ Topiramate, 50 mg orally daily.

¶ Phentermine-topiramate, 7.5-46 mg orally daily.

### Adverse events

Five postoperative adverse events occurred in 4 patients (80%), including transient acute kidney injury (n = 2) that resolved with increased home fluid intake, vomiting (n = 1) that resolved with antiemetics, epigastric pain (n = 1) that resolved with pain medication, and esophageal candidiasis that resolved with fluconazole (n = 1) (Table 2). Adverse events were all mild, with none exceeding grade I on the Clavien-Dindo scale. No patient required emergency department visit or hospitalization for postoperative adverse events. No patient missed any doses of immunosuppressive medications; all patients were on tacrolimus, and trough levels remained stable and within therapeutic range during follow-up (Table 4). Liver graft function remained stable without evidence of rejection.

**Table 4:** Tacrolimus levels and hepatic function tests∗

	Case 1	Case 2	Case 3	Case 4	Case 5
Tacrolimus trough level					
Pre-op, ng/mL	4	7.6	5.4	7.9	8.4
Post-op, ng/mL	7	7.7	8	10	8
Hepatic function tests					
Pre-op					
T bili, mg/dL	0.2	0.3	0.5	0.2	0.4
AST, U/L	36	20	20	31	24
ALT, U/L	48	25	20	70	38
Post-op					
T bili , mg/dL	0.3	0.4	0.4	0.4	0.4
AST, U/L	37	24	24	20	21
ALT, U/L	55	21	23	20	25

*ALT*, Alanine transaminase; *AST*, *aspartate* transaminase; *T bili*, total bilirubin.

∗ All patients had post-op laboratory tests within 1 month of the procedure.

## Discussion

Obesity after LT can compromise graft health and patient outcomes, and there are minimal data on optimal weight loss methods after LT. We hypothesized that ESG presents multiple advantages over other weight loss interventions, including its strong safety profile and avoidance of adherence issues with, and minimizing potential drug interactions between, post-LT medications and AOMs. ESG also facilitates uninterrupted administration of oral immunosuppressive medications, unlike metabolic surgery.

To our knowledge, this is the first case series demonstrating the feasibility of ESG after LT. Adverse events were mild and resolved with minimal intervention. Calcineurin inhibitor trough levels remained stable and within therapeutic range, with no cases of graft rejection during follow-up. Our data also show the efficacy of ESG after LT, with median TBWL of 27.2%. Three patients achieved TBWL >20%, which exceeds the average expected TBWL.^5^ We attribute this to improved suture durability with the use of more bites per suture, selection of highly motivated patients, and concomitant AOM use, although most patients terminated AOM use after ESG. Notably, 2 patients experienced weight loss plateau. Both of those patients were lost to follow-up with gastroenterology and nutrition visits for at least 4 months, which is an important determinant of weight loss plateau. These findings suggest that optimal patient selection and close follow-up are crucial for successful outcomes.

We incorporated some modifications to the standard procedural technique and peri-procedural management. First, although using 6 to 8 bites per suture is typical,^9^ we found that using more bites per suture helps distribute the tension across multiple contact points, leading to lower risk of suture breakage without increasing overall load on each suture. Some centers use a U-shaped suturing pattern, based on data showing superior reduction in gastric capacity. However, we think that our technique effectively maintains the narrowed gastric lumen without creating additional tensile stress that has been described with the U-shaped pattern.^10^ In addition, given the theoretically increased infection risk with full-thickness suturing in post-LT patients on immunosuppressants, we recommended a course of antibiotics postoperatively for primary prophylaxis.

Limitations of our study include small sample size, and significant heterogeneity in time between LT and ESG (range, 484 to 4500 days; mean, 1892 ± 1562 days), AOM use, and clinic follow-up rates.

Our findings support the use of ESG after LT, but further research is needed to validate these results and refine patient selection criteria. Given the small sample size and retrospective design of our study, these findings should serve as foundation for larger, prospective clinical trials rather than for clinical application. Future studies should also investigate alternative bariatric modalities, such as endoscopic balloon placement and duodenal ablation, in the post-LT population.

## Disclosure

All authors disclosed no financial relationships. K.E. Lunsford receives unrelated National Institutes of Health grant funding from R21AI180739, R01DK137222, and R44HL172564.
